# Evaluation of heart rate variability and behavior of electrocardiographic parameters in dogs affected by chronic Monocytic Ehrlichiosis

**DOI:** 10.1371/journal.pone.0216552

**Published:** 2019-05-24

**Authors:** Mauricio Gianfranchesco Filippi, Mayra de Castro Ferreira Lima, Antonio Carlos Paes, Amanda Sarita Cruz Aleixo, Eunice Oba, Fabiana Ferreira de Souza, Regina Kiomi Takahira, Maria Lucia Gomes Lourenço

**Affiliations:** 1 Department of Veterinary Clinics, São Paulo State University (Unesp), School of Veterinary Medicine and Animal Science, Botucatu, São Paulo, Brazil; 2 Department of Veterinary Hygiene and Public Health, São Paulo State University (Unesp), School of Veterinary Medicine and Animal Science, Botucatu, São Paulo, Brazil; 3 Department of Animal Reproduction and Veterinary Radiology, São Paulo State University (Unesp), School of Veterinary Medicine and Animal Science, Botucatu, São Paulo, Brazil; University of Bologna, ITALY

## Abstract

Canine Monocytic Ehrlichiosis (CME) is a systemic disease prevalent in the entire world caused by the obligate intracellular bacteria *Ehrlichia canis*. The occurrence of myocarditis with a high prevalence of arrhythmias in dogs affected by this disease in the cytopenic phase has already been proven. This study aims to evaluate the concentrations of CK MB, cTnI and NT-proBNP in dogs affected by Ehrlichia canis in the chronic phase since the intense stimulation of the immune system can lead to myocarditis; to evaluate if the condition can lead to arrhythmic events and, if so, define their frequency and classification through conventional and ambulatory electrocardiogram tests (Holter method) for a period of 24 hours; to analyze heart rate variability in the time domain and whether the condition can lead to autonomic imbalance; and to determine the survival rate of affected dogs, identifying possible risk factors for mortality at this stage of the disease. For this purposes, we evaluated clinical, hematological and biochemical data, as well as the concentrations of cardiac biomarkers Creatine Kinase-MB (CK MB), Cardiac Troponin I (cTnI) and N-terminal pro-peptide natriuretic type B (NT-proBNP). We also analyzed conventional and ambulatory electrocardiography (24-hour Holter) and heart rate variability (HRV) in 20 dogs afflicted by cytopenic CME in the chronic phase of the disease (G1) and compared the results with a control group comprised of ten healthy dogs (G2). G1 was monitored during the treatment for 28 days, during which eight (8) of the 20 infected dogs died (40%). Anorexia, vomiting, fatigue, hypoalbuminemia, heart murmurs and increased concentrations of alanine aminotransferase (ALT) and alkaline phosphatase (ALP) were common clinical signs. The mean concentrations of cTnI and CKMB were significant (0.24 ng / mL ± 0.5, 229 ± 205 IU / mL) in comparison to the control group (0.042 ± 0.07 ng / mL, 126 ± 46.12 IU / mL). No significant differences were observed between NT-proBNP concentrations in G1 (135.46 ± 29.7) and G2 (138.28 ± 19.77). Nine of the twenty dogs (45%) presented a high frequency of arrhythmias during 24-hour recording, ranging from first and second-degree atrioventricular block, ventricular and supraventricular ectopic events and sinus tachycardia. No sinus pause was observed. One dog had 120 episodes of unsustained ventricular tachycardia and two episodes of sustained ventricular tachycardia. The short-term and long-term HRV data, represented by SDNN (ms), SDANN (ms) and pnn50 (%) were also significant lower (83 ± 65, 56.05 ± 37.3 and 14.56 ± 20, respectively) in comparison to the healthy animals (268 ± 74.6, 168.3 ± 39.14 and 55.87 ± 12.8, respectively). These results suggest that cytopenic CME is characterized by an arrhythmogenic component and intense stimulation of the sympathetic autonomic nervous system in the heart, reflecting an imbalance in the activity of the ANS.

## Introduction

Canine Monocytic Ehrlichiosis (CME) is an infectious disease distributed worldwide caused by the obligate intracellular bacteria *Ehrlichia canis* and transmitted by the tick *Rhipicephalus sanguineus*. The cytopenic phase of the disease is the most serious, with high mortality rates. It is still not fully understood which factors trigger it, but the most commonly accepted theory implicates the involvement of a possible immune disorder [1,2].

Ehrlichia uses evasion mechanisms to multiply and spread systemically, causing a strong inflammatory response that is often ineffective at clearing these stealthy pathogens. Persistent intravascular infection can cause chronic inflammation and vasculitis, leading to injuries in multiple organs [3,4].

Apathy, anorexia, splenomegaly, hemorrhagic tendencies, pancytopenia, hypoalbuminemia, hyperglobulinemia and increased serum concentration of alanine aminotransferase (ALT) are frequent changes observed on dogs at this stage [5]. Myocarditis is also common in cases of CME, similarly to other infectious diseases such as Babesiosis, Parvovirus, Chagas Disease, Leishmaniosis and Leptospirosis [6–10]. It is an inflammation of the cardiac muscle that may potentially damage the myocardial contraction and progress to dilated cardiomyopathy and chronic congestive heart failure [11,12]. It has been shown that naturally infected dogs in the acute phase of Monocytic Ehrlichiosis present a high incidence of rhythm and conduction anomalies in conventional ECG tests, but the severity of these arrhythmias remain unknown [13].

This study aims to evaluate the concentrations of CK MB, cTnI and NT-proBNP in dogs affected by Ehrlichia canis in the chronic phase since the intense stimulation of the immune system can lead to myocarditis; to evaluate if the condition can lead to arrhythmic events and, if so, define their frequency and classification through conventional and ambulatory electrocardiogram tests (Holter method) for a period of 24 hours; to analyze heart rate variability in the time domain and whether the condition can lead to autonomic imbalance; and to determine the survival rate of affected dogs, identifying possible risk factors for mortality at this stage of the disease.

## Materials and methods

### Animals

Twenty sick dogs treated in the Department of Veterinary Hygiene and Public Health of the Veterinary Hospital maintained by the College of Veterinary Medicine and Animal Sciences, São Paulo State University (FMVZ UNESP) in the city of Botucatu, Brazil, between July 2014 and July 2016, were selected for this study (G1). The animals were of different breeds, were five years-old and presented various body weights.

The inclusion criteria adopted for the study followed a previous classification (Mylonakis et al., 2004): (1) Clinical signs such as depression, anorexia, splenomegaly, bleeding and history of tick infestation; (2) hematologic changes indicating bone marrow hypoplasia, such as non-regenerative anemia (PCV ≤ 25%), leukopenia (WBC ≤ 3,000 cells/μL) with reduction of all cell lines and severe thrombocytopenia (platelet count < 20,000/μL); (3) positive nested PCR assay for *Ehrlichia canis* in the whole blood sample. The animals were tested only for *Ehrlichia canis*.

The exclusion criteria adopted were: dogs over five years-old, dogs undergoing treatments with antibiotics that destroy E. canis (Tetracyclines or Chloramphenicol), dogs with history of heart diseases detected on echocardiogram, including valve diseases.

Ten healthy animals, kindly provided by their owners, served as the control group (G2). The criteria for dogs to be considered healthy were normal results in the blood pressure test, blood tests, physical examination and cardiopulmonary auscultation; absence of valvular diseases on the echocardiogram; normal recording for five minutes on conventional ECG and 24-hour Holter; and a clean health history verified with the owners.

The proposed exams were conducted in the same order for all 30 dogs: determination of arterial blood pressure, physical examination, precise cardiopulmonary auscultation, blood collection, five-minute recording in conventional ECG and 24-hour Holter monitoring, and also by the anamnesis held with the owners.

An echocardiogram was performed only for identify valvular diseases and exclude those individuals from the study.

Control animals presenting changes in any of these parameters were excluded from the study. The Ethics Committee on the Use of Animals (CEUA-FMVZ-Unesp Botucatu) approved the study under protocol number 83/2014 as being in accordance with the ethical principles for animal trials. Owners signed an informed consent form to authorize the animal’s participation in the study.

In addition, the tests and exams proposed in this study were performed prior to the start of treatment for the condition, after confirming the diagnosis by the PCR method. The animals received follow-up care at the hospital by the veterinarian that originally treated them at admission and contact with the authors of this study was maintained because the data regarding animals finishing the treatment was compiled for a future evaluation of the survival curve.

### Systolic arterial blood pressure measurement

Non-invasive systolic blood pressure measurement was the first test conducted to minimize stress-related factors and avoid overestimating values. The equipment used was a vascular Doppler ultrasound device (Parks model 811-B) following previously proposed techniques [14–16].

The blood pressure cuff was attached to the left thoracic limb at the distal portion of the radius, not exceeding the dotted lines indicated on the cuff itself. To improve the accuracy of the reading, the appropriate cuff was chosen according to the size of the dog’s limb (40% of the diameter of the limb where the measurement was performed), and the final value considered was the arithmetic mean of three measurements.

### Collection and analysis of biological material

Ten milliliters of blood was collected from each animal through jugular venipuncture. The collected material was immediately added to a tube containing EDTA-K2 as an anticoagulant agent for hematologic and for the molecular analysis another tube containing a coagulation activator and determination of CK-MB, cTnI, NT-proBNP, blood urea nitrogen (BUN), creatinine, alanine and aspartate aminotransferase (ALT and AST), alkaline phosphatase (ALP), total protein and total albumin levels.

The hematological analysis was performed as follows: (1) Plasma Cell Volume (PCV) through the microhematocrit technique, (2) total plasma protein concentration through the plasma refractometry technique, (3) total count of red blood cells and white blood cells with electronic cell counter (pocH-100iV) and leukocyte differentiation through 100-cell differential leukocyte count, (4) platelet count in Neubauer Chamber with 1% ammonium oxalate as diluent. The blood smear was also verified for the presence of *Ehrlichia canis*.

Blood samples for biochemical assays were centrifuged for 10 minutes at 1532 G. The biochemical analyses were done on a Biochemical Automatic Analyzer (Cobas Mira Plus, Roche).

#### Molecular diagnosis

The molecular diagnosis was conducted by PCR (Polymerase chain reaction) in a private lab (VETDNA) in Botucatu–SP. The PCR technique used to detect *Ehrlichia canis* was the *nested* PCR technique described in a previous study [16]. It consists of two steps: 1) Amplifying bacteria belonging to genus *Ehrlichia* (*E*. *canis*, *E*. *chaffeensis*, *E*. *muris*) and *Neorickettsia helminthoeca* when present on the blood sample through ECC and ECB specific primers. 2) Differentiating *E*. *canis* from the other members of the genus by repeating the same technique with different primers and DNA templates, HE-3/ECA.

#### Concentration of CK-MB

Serum concentrations of CK-MB were determined using the kinetic-UV method with a commercially available kit (CK-MB-1–UV–K069, Bioclin) in an automatic biochemical analyzer (Cobas Mira Plus, Roche).

#### Concentration of cardiac troponin I (cTnI)

Concentrations of cardiac troponin were determined in an automated immunoassay analyzer (mini-VIDAS, Biomeriéux). The technique used was the ELFA (Enzyme-Linked Fluorescent Assay) technique.

#### Concentration of NT-proBNP

Concentrations of NT-proBNP were determined using the enzyme immunoassay method (sandwich type) for N-terminal pro–B-type natriuretic peptide (Cloud Clone Corp). The absorbance plate was read at 450 nm.

### Conventional electrocardiographic evaluation

The conventional ECG exam was performed with a 12-channel digital electrocardiograph (ECGPCVet, TEB) calibrated to 1 mV/cm and 50 mm/s. The exam was performed with the animal in lateral recumbency following the previously described to the technique [17,18]. The analysis was performed on a standard 10-lead ECG (DI, DII, DIII, aVR, aVL, aVF, rV2, V2, V4, and V10) for five minutes. Analysis of wave amplitude (mV) and duration (ms) was conducted on lead DII. Base rhythm, heart rate and mean electrical axis of the QRS complex were the main parameters obtained [19,20]. Rhythm and conduction disturbances were registered and classified separately.

### Ambulatory electrocardiographic monitoring (holter system)

Holter Monitoring data was recorded with a Cardiolight three-channel digital device (Cardios, SP, Brazil). The four electrodes were positioned in a horizontal plane around the fifth intercostal space on both sides of the thorax and the data obtained was analyzed automatically by a Cardiosmart 550 device (Cardios, SP, Brazil). Corrections were made manually. The main parameters evaluated were base rhythm; minimum, mean and maximal heart rate; pauses longer than two seconds; presence of abnormalities; and supraventricular and ventricular ectopic events, if any. The accepted artifact threshold was lower than 5%.

#### Heart rate variability (HRV)

The time-domain HRV parameters analyzed were: (1) standard deviation of all RR intervals (SDNN), (2) standard deviation of the average RR interval taken every five minutes (SDANN), (3) mean standard deviation of RR intervals taken every five minutes (SDNN INDX), (4) root mean square of the successive differences (rMSSD) and (5) the percentage of differences exceeding 50 ms between adjacent RR intervals (pNN50).

### Statistical analysis

For quantitative data such as systolic blood pressure, electrocardiographic parameters and values obtained for time-domain heart rate variability, data normality was evaluated with a Shapiro-Wilk test. For quantitative data such as hematological, biochemical and biomarker parameters, the study employed the Kruskal-Wallis test to determine if the data followed a Gaussian distribution. For parametric and non-parametric values, the study employed, respectively, a t-test for independent samples and a Mann-Whitney test to detect statistically significant differences between the sick (G1) and the control (G2) groups. For the qualitative data obtained from the medical history and physical examinations of G1 animals, the study employed a descriptive analysis and Fisher’s exact test to compare the relative frequencies of each characteristic between animals, including both those that survived the entire 28-day monitoring and those that died during the period. Kaplan-Meier survival curves were plotted to compare, under various conditions, the G1 animals that died and those that survived during the 28-day follow-up. A log-ranked test was developed to detect statistically significant differences between the curves. The significance level adopted was p < 0.05.

## Results

### Animals

Twenty sick animals were evaluated, ten males and ten females, eight of which were pure breeds including Labrador Retrievers, Yorkshire Terriers, Pugs, Schnauzers, Lhasa Apsos, Border Collies, Bull Terriers, and Pitbulls, while twelve were mixed breeds. The mean and range for age (years) and body weight (kg) were 2.7 (0.4–6) and 11.17 (2.6–24.8) respectively. Of the ten animals evaluated in the control group, comprised of five males, and five females, seven were pure breeds, including Labrador Retrievers, Cocker Spaniels, Yorkshire Terriers, Border Collies, Pitbulls, German Shepherds, Australian Collies, while three were mixed breeds. The mean and range for age (years) and body weight (kg) were 1.5 (0.75–5) and 23.01 (4.5–35) respectively.

#### Clinical parameters

**Fig 1:**
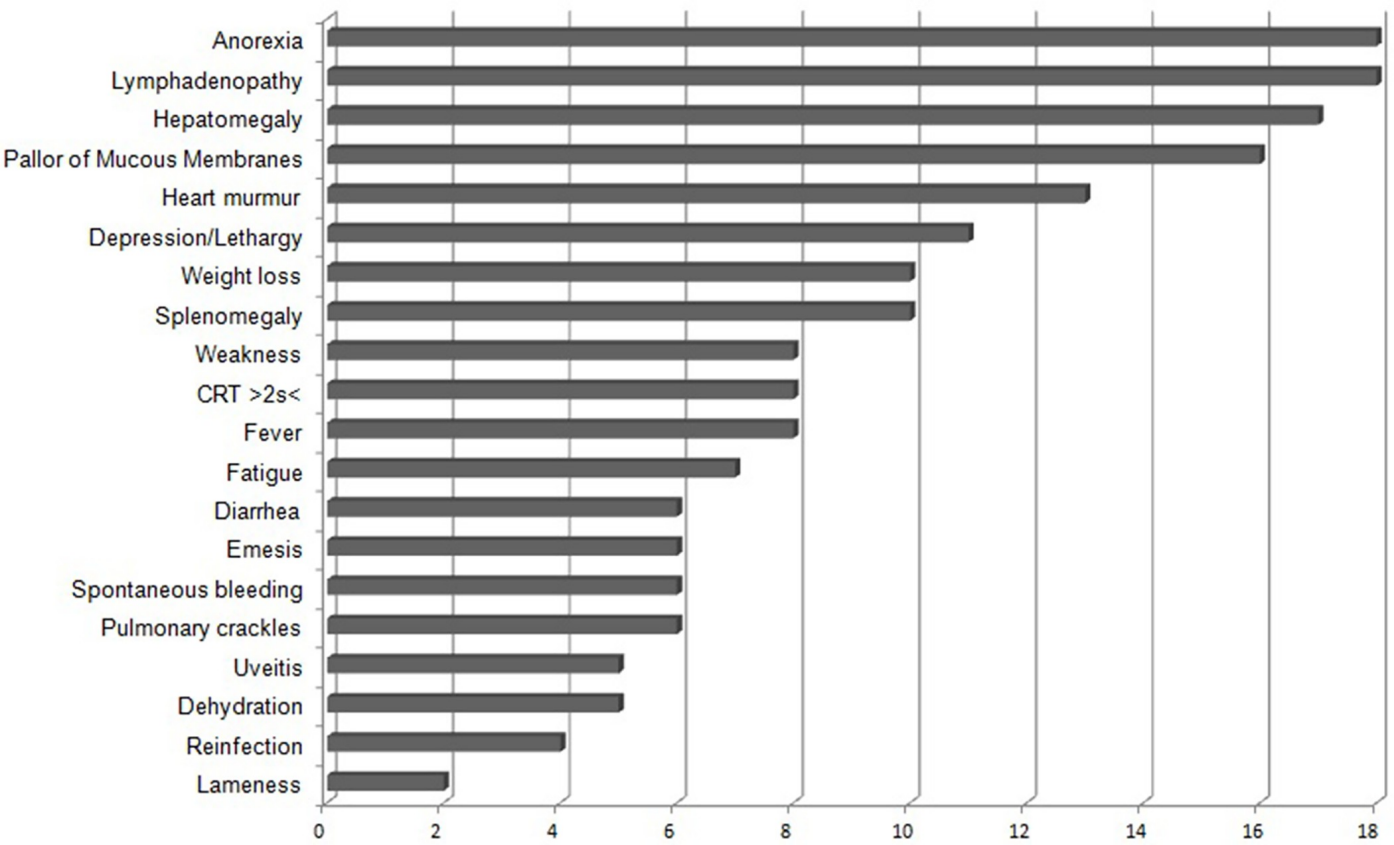
Relative frequency of clinical signs and pathological conditions in 20 natural cases of Chronic Canine Ehrlichiosis (G1).

Medical history and physical examination data for G1 animals were collected during the consultation. The distribution of absolute frequencies is shown in Fig 1.

**Fig 2:**
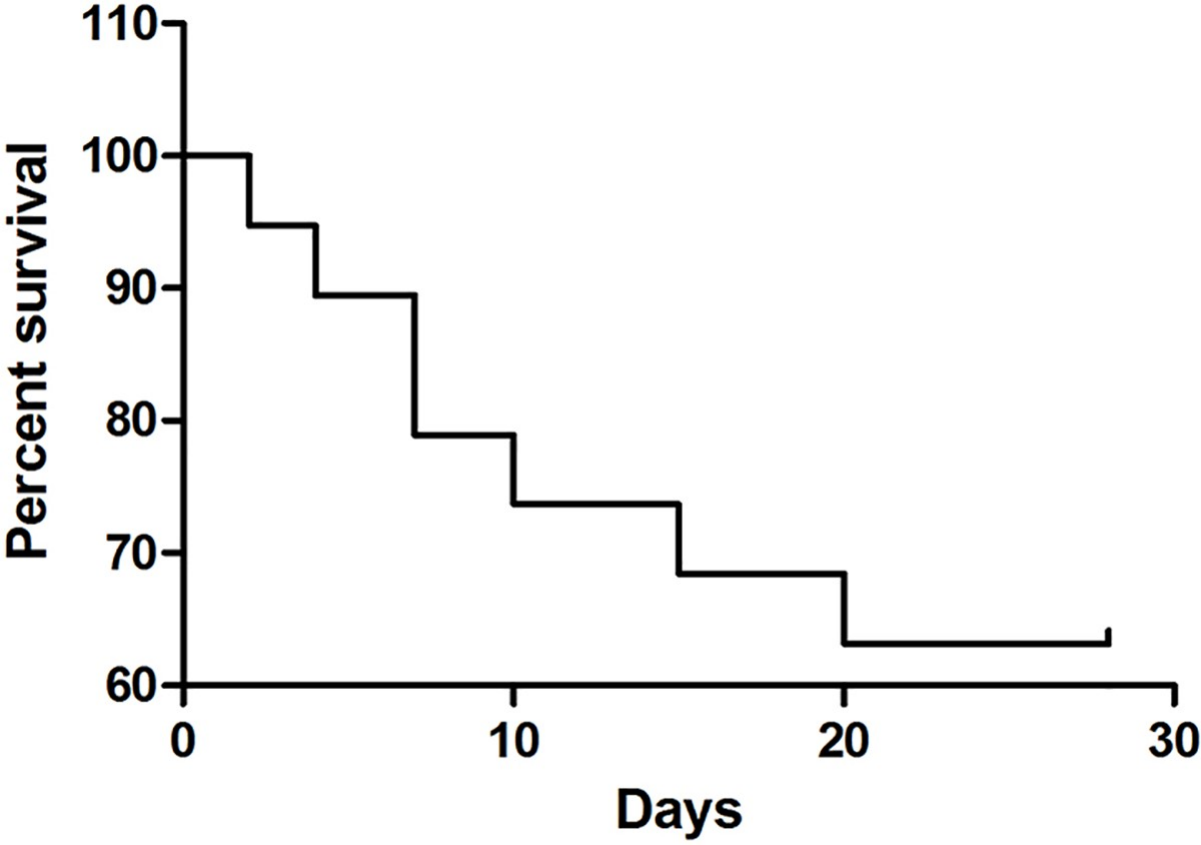
Survival analysis of the sick group (G1) after a 28-day monitoring period.

Owners of animals with cytopenic CME often reported anorexia (15 animals), weight loss (nine animals), fatigue (seven animals), weakness (seven animals), bleeding episodes (six animals), as well as ophthalmologic (five animals) and articular (two animals) injuries. There were no complaints of coughing or sneezing. Only four animals had contracted this disease previously (over a year prior). The survival curve for G1 animals is shown in Fig 2.

The same procedure was followed for the physical examination data. High relative frequencies could be observed across the board: 11 of the 20 dogs were apathetic, and eight dogs presented high capillary refill times (CRT). Five animals presented CRT values of one second and another three presented values between one and three seconds. Eighteen dogs presented moderate increases in one or more lymph nodes.

Cardiac auscultation revealed a high incidence of alterations: 13/20 dogs (65%) presented at least a grade III or IV/VI holosystolic murmur with mitral and tricuspid focus. S3 heart sounds were detected in three cases (15%). Pulmonary crackles were noticed in six animals (30%), mainly in the cranial lobes.

Data from abdominal palpation revealed alterations in 17 of the 20 dogs (85%) studied. In most cases, they presented organomegaly in the ventral epigastric region, suggesting hepatomegaly or splenomegaly. Another common alteration observed was abdominal pain.

The body condition of the dogs was also evaluated subjectively and revealed that 10 animals presented a body score of 2/9, considered too skinny. The quantitative variables from the physical examination are shown in Table 1. Significant differences were observed for the heart rate parameters, and the respiratory rate was the same for both groups.

**Table 1:** Mean and standard deviation of the clinical parameters analyzed for both groups and the results of the statistical test.

Clinical Parameters	Sick Group	Control Group	p value
**Heart rate (bpm)**	142.1 ± 32.4	110 ± 20.5	0.0103^a^*
**Respiratory rate (mpm)**	83.2 ± 58.9	47.75 ± 43.14	0.1196^b^

^a^ t-test for independent samples

^b^ Mann-Whitney test; Significant differences (*) are expressed in the horizontal lines and the superscript letters indicate the test used.

Statistical significance: p<0.05

#### Hematological parameters

The quantitative hematological variables for both groups are shown in Table 2. The results were significantly lower for all parameters in comparison to the healthy animals. Kaplan-Meier curves were plotted for all parameters. Curves relating the monocyte and platelet counts diverged subjectively, but the p values observed in the log-ranked test were 0.26 and 0.17, respectively.

**Table 2:** Mean and standard deviation of hematological parameters analyzed for both groups and the results of the statistical test.

Hematological Parameters	Sick Group	Control Group	p-value	Reference ^21^
**Red Blood cells (x 10**^**6**^**/mm**^**3**^**)**	2.97 ± 0.28	7.48 ± 0.67	< 0.0001*^b^	5.5–8.5
**Hemoglobin (g/dL)**	6.4 ± 2.62	16.98 ± 1.24	< 0.0001*^b^	12.0–18.0
**PCV (%)**	19.8 ± 7.71	47.1 ± 3.38	< 0.0001*^b^	37.0–55.0
**VCM (fl)**	68.18 ± 8.1	63.17 ± 4.1	0.08^a^	60.0–77.0
**CHCM (g/dL)**	32.11 ± 1.7	36.08 ± 1.89	< 0.0001*^a^	32.0–36.0
**RDW (%)**	16.57 ± 3	11.17 ± 1.16	< 0.0001*^b^	12.0–15.0
**Platelet Count (x 10**^**3**^**/μL)**	11.52 ± 9.35	271 ± 84.84	< 0.0001*^b^	160.0–430.0
**White Blood cells (x 10**^**3**^**/μL)**	1.5 ± 2.09	10.97 ± 2.10	<0.0001*^b^	6.0–17.0
**Neutrophils (x 10**^**3**^**/μL)**	1.17 ± 1.6	6.75 ± 1.48	< 0.0001*^b^	0–0.3
**Lymphocytes (x 10**^**3**^**/μL)**	0.19 ± 0.39	2.41 ± 0.69	< 0.0001*^b^	1.0–4.8
**Eosinophils (x 10**^**3**^**/μL)**	0.01 + 0.04	1.1 ± 0.64	< 0.0001*^b^	0.1–1.25
**Monocytes (x 10**^**3**^**/μL)**	0.12 ± 0.2	0.69 ± 0.4	< 0.0001*^b^	0.15–1.35

^a^ t-test for independent samples

^b^ Mann-Whitney test

*Statistical significance: p<0.05

#### Biochemical parameters

The results of the biochemical analysis for both groups, including the concentrations of biomarkers, are shown in Table 3.

**Table 3:** Mean and standard deviation of biochemical parameters analyzed of both groups and the results of the statistical test.

Biochemical Parameters	Sick Group	Control Group	p-value	Reference^22^
**Albumin (g/dL)**	2.1 ± 0.54	3.5 ± 0.27	< 0.0001 ^a^*	2.6–3.3
**Total protein (g/dL)**	6.56±1.45	6.8±0.58	0.39^b^	6.0–8.0
**Globulin (g/dL)**	3.67 ± 1.55	2.58 ± 0.44	0.0396 ^a^*	2.7–4.4
**ALT (IU/L)**	93.95 ± 82.7	41.7 ± 6.9	0.213^a^	21–73
**ALP (IU/L)**	174.45 ± 202.83	49.7 ± 44.2	0.0004 ^a^*	20–156
**GGT (IU/L)**	2.06 ± 4.4	1.34 ± 0.95	0.7132^a^	1.2–6.4
**Urea (mg/dL)**	47.07 ± 27.36	35.15 ± 9.56	0.618^a^	21.4–59.9
**Creatinine (mg/dL)**	0.78 ± 0.36	0.9 ± 0.28	0.359^a^	0.5–1.5
**CKMB(IU/L)**	229 ± 205.9	126 ± 46.12	0.0408 ^a^*	47–184^[^^22^^]^
**NT-ProBNP (pmol/L)**	135.46 ± 29.7	138.28 ± 19.77	0.46^b^	403–980^[^^23^^]^
**Cardiac Troponin I (ng/dL)**	0.2493 ± 0.50	0.042 ± 0.08	0.05 ^a^*	≤ 0.11^[^^24^^]^

^a^ Mann-Whitney test

^b^ t-test for independent samples

*Statistical significance: p < 0.05

The albumin and globulin analyses revealed significantly lower and higher values, respectively, in comparison with the control group. The data revealed that chronically infected animals presented hypoalbuminemia and hyperglobulinemia, with concentrations between 1.1–2.9 and 1.6–7.4 g/dL, respectively.

Regarding the hepatic function enzymes, the ALT and ALP values were, on average, higher than the reference range (30 and 40%, respectively), but only the second one presented a statistically significant difference when compared to G2.

Markers for renal function were within the reference values for the species for most animals in both groups, except for five dogs (25%) in the SG that presented increased BUN values, with a maximum of 107 mg / dL. For the creatinine values, four dogs (20%) presented low concentrations. In both cases, there was no statistically significant difference in comparison with G2.

**Fig 3:**
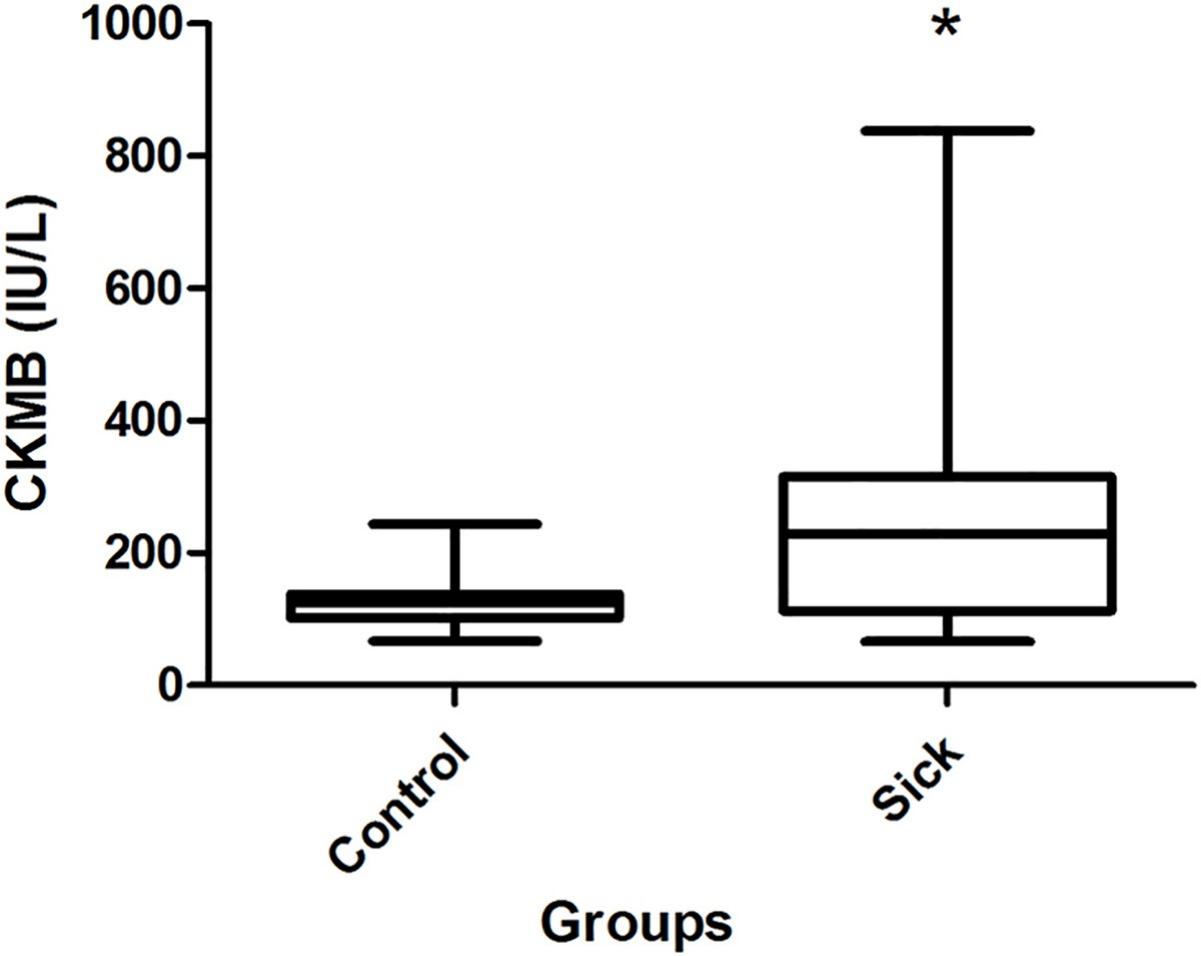
Sample distribution of CK-MB (UI/L) concentrations between the groups with the results of the statistical test.

Regarding the cardiac biomarkers, the concentration of CKMB in G1 was, on average, higher than the reference values in 11/20 (55%) dogs and presented statistically significant differences when compared to G2 (p = 0.04). Sample distribution for the values is presented in Fig 3.

**Fig 4:**
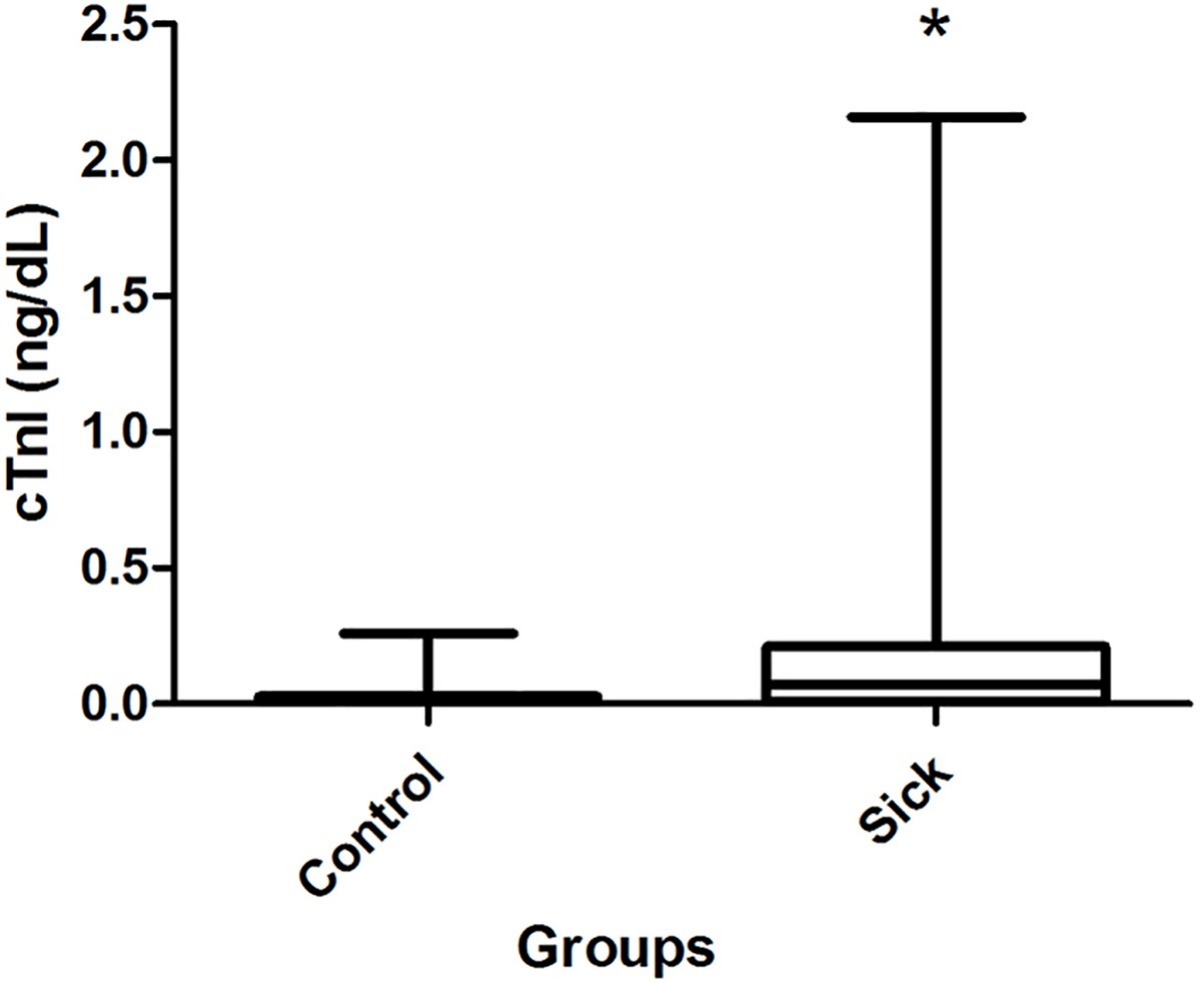
Sample distribution of cardiac Troponin I (ng/dL) concentrations between the groups with the results of the statistical test.

As for the concentrations of cTnI, G1 animals presented increased concentrations in seven (35%) cases, and the values were significantly higher than in G2 (Fig 4). Extreme concentrations were recorded in a few cases, such as 0.46, 0.9 and 2.14 ng/mL. The survival analysis compared dogs with high and low concentrations of cTnI, but no significant difference was observed between the curves.

**Fig 5:**
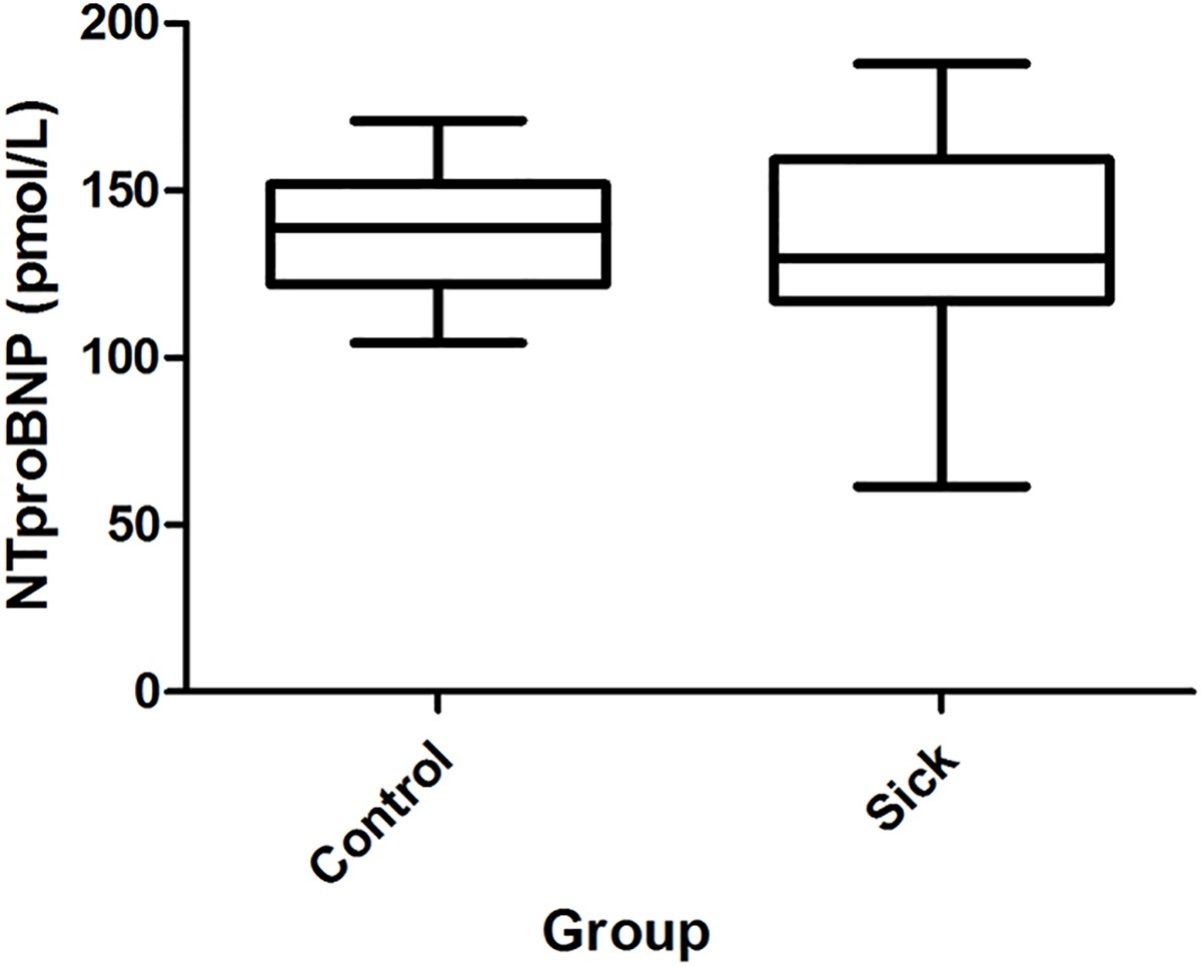
Sample distribution of NT-proBNP concentrations between the groups with the result of the statistical test.

Values for NT-proBNP are presented in Fig 5. The results were within the reference values in G1, and no significant differences were observed between G1 and G2.

#### Blood pressure analysis and conventional ECG

The mean and standard deviation (SD) for the non-invasive systolic blood pressure test (mmHg) were 121.93 ± 20.2 and 142.5 ± 14.57 respectively for G1 and G2. The statistical test showed a significant difference (*p* = 0.01).

**Fig 6:**
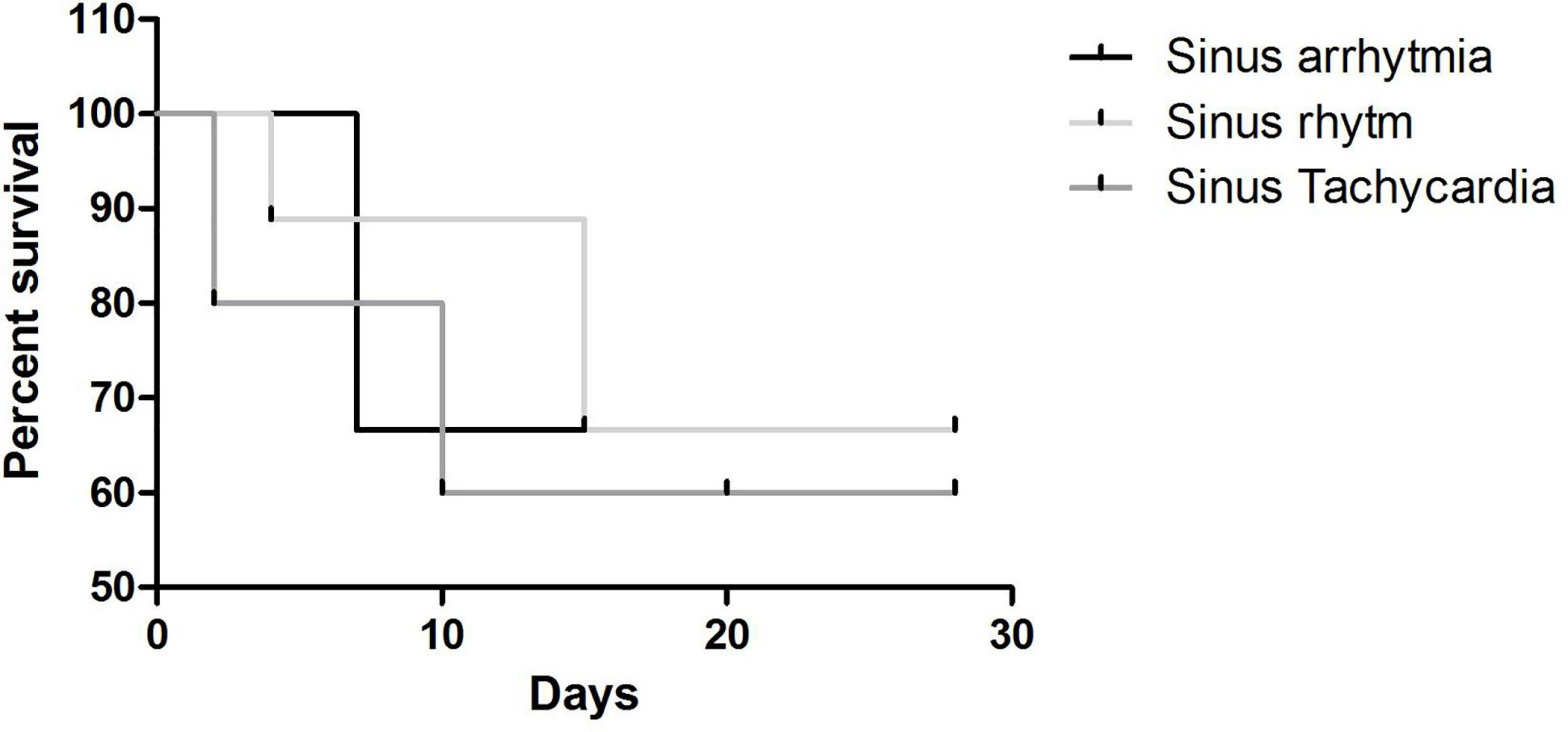
Survival analysis chart according to the base rhythm observed in the conventional ECG examination for the sick group (G1).

**Fig 7:**
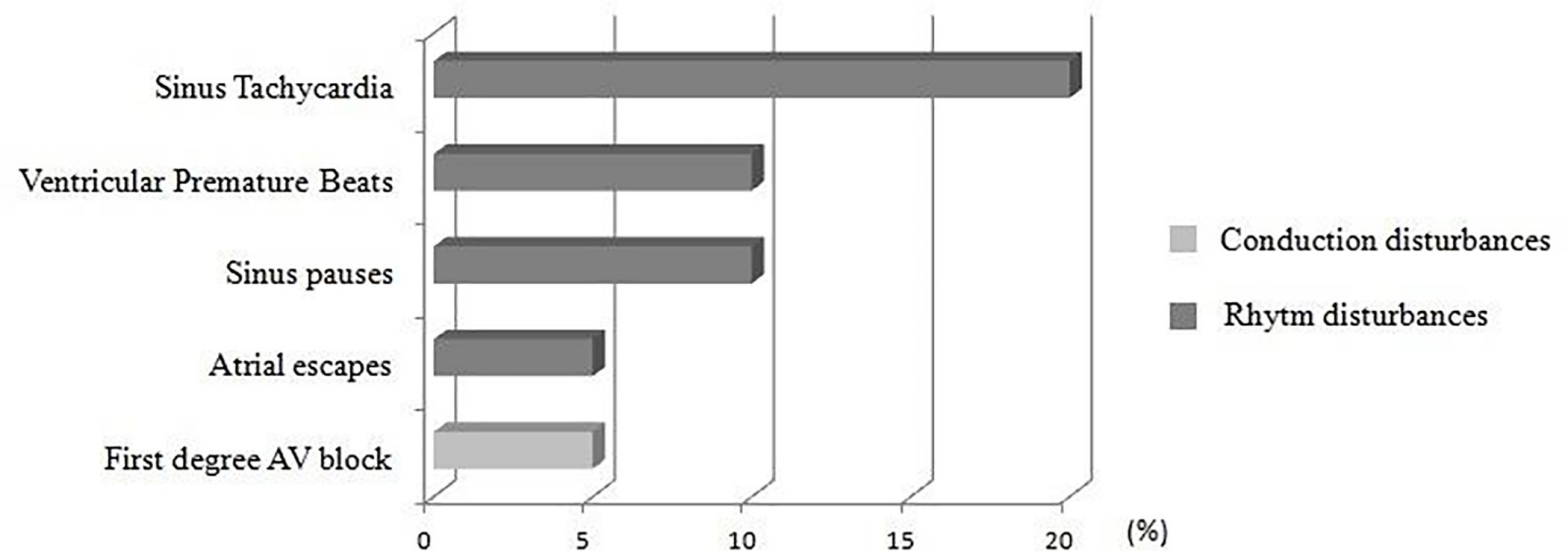
Chart showing the frequency of ECG changes in dogs with chronic Monocytic Ehrlichiosis over five minutes.

The predominant rhythms in the ECG were sinus arrhythmia, sinus rhythm and sinus tachycardia (30%, 45%, and 25% respectively on G1 and 70%, 30% and 0% respectively on G2). Sinus arrhythmia was the predominant rhythm in animals of the control group (70%), which differed from the sick group. Therefore, a survival curve was plotted for the animals according to ECG rhythm, and the results are presented in Fig 6. Subjectively, sinus tachycardia was revealed as a possible factor for increased mortality risk due to the high incidence in G1 animals during the 28-day follow-up (3/8). However, this result was not statistically significant (*p* = 0.31). Nine dogs (45%) presented arrhythmias in the conventional ECG examination. Fig 7 exemplifies the frequency of the alterations observed.

The relationship between duration and amplitude of the ECG waves is presented in Table 4. Although the heart rate of G1 animals is subjectively higher when compared to G2 animals, it has not been shown to be statistically significant in the sample distributions. No values exceeded the reference values, except for two dogs with high PR interval (> 130 ms) due to a first-degree atrioventricular blockage. However, the amplitudes of P and R waves were significantly lower in G1 than in G2.

**Table 4:** Mean and standard deviation of electrocardiographic parameters analyzed in the sick and control groups. P values represent the results of the statistical tests.

Parameters	Sick Group	Control Group	P value	Reference
**Heart Rate ECG**	138.11 ± 31.27	125.5 ± 16.5	0.2864^a^	70–160
**Mean Electrical Axis**	67.1 ± 20.3	61 ± 3.16	0.286^a^	40–100
**P wave–Duration**	48.6 ± 3.60	49.3 ± 3.68	0.567^a^	30–50
**PR Interval**	96.2 ± 4.47	100.6 ± 5	0.5496^b^	60–130
**QRS complex–Duration**	54.95 ± 6.56	56.3 ± 3.77	0.5538^b^	Up to 50
**QT Interval**	196.4 ± 27	206.7 ± 8.7	0.248^a^	150–250
**P wave–Amplitude**	0.148 ± 0.04	0.2 ± 0.04	0.00024*^b^	<0.4
**Q wave–Amplitude**	0.152 ± 0.13	0.243 ± 0.2	0.328^a^	0–0.5
**R wave–Amplitude**	0.97 ± 0.44	1.14 ± 0.18	0.035*^a^	0.5–2.5
**S wave–Amplitude**	0.135 ± 0.2	0.088 ± 0.05	0.845^a^	0–0.3
**ST segment Depression**	0.054 ± 0.04	0.048 ± 0.45	0.73^b^	< 0.15
**T wave–Amplitude**	0.02 ± 0.16	0.005 ± 0.09	0.6608^b^	<25% R Wave

^a^ Mann-Whitney test

^b^ t-test for independent samples.

*Statistical significance: p<0.05

Ventricular repolarization analysis revealed no ST segment abnormalities such as elevations or depressions in the G1 exceeding the standard limit. The values also did not show statistically significant differences when compared to the control. The amplitude of the T wave was increased in 10 (50%) of the sick group dogs, in contrast with one dog (10%) in the control group. Fischer's exact test revealed this difference to be statistically significant (*p* = 0.048).

#### Holter and heart rate variability (HRV) analysis

**Fig 8:**
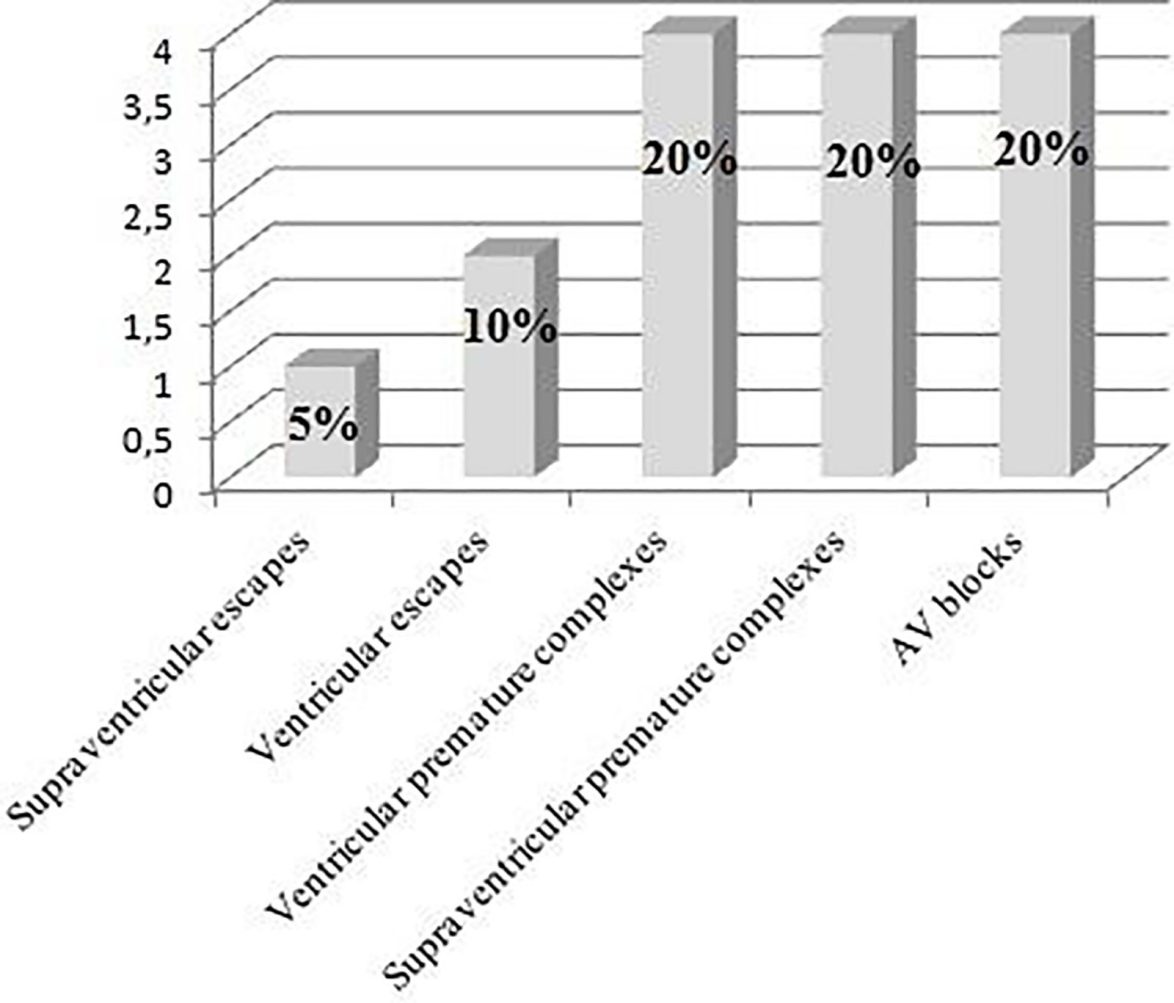
Chart showing the prevalence of arrhythmias during 24-hour recording for G1.

**Fig 9:**
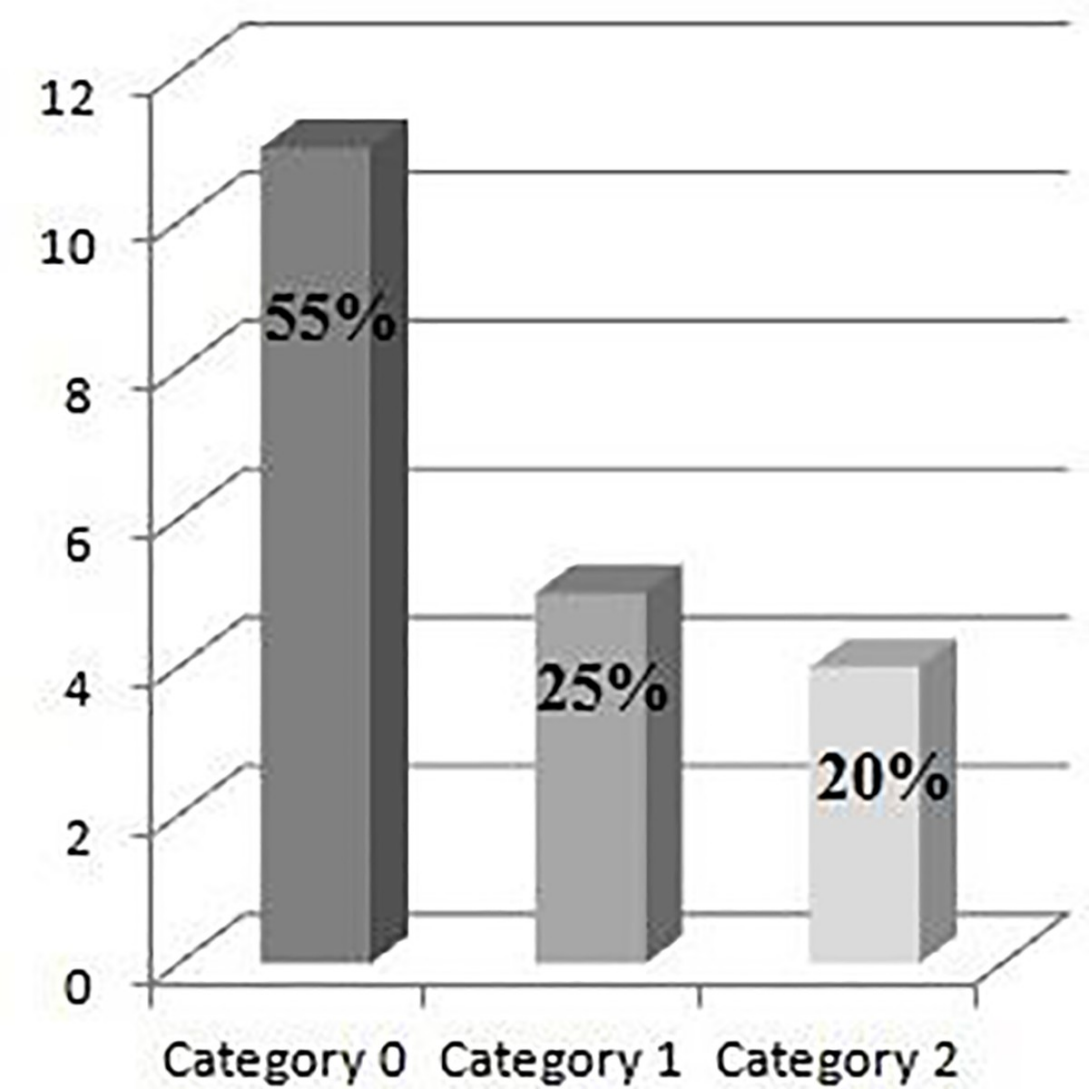
Chart showing the prevalence of arrhythmias in the G1 according to the categories established on the Holter exam.

Nine dogs in G1 presented arrhythmias. Their exact classifications are presented in Fig 8. For the quantitative analysis, the animals were classified into three categories (Fig 9): Category 0: presence of 15 isolated events or pairs. Absence of ventricular tachycardia or supraventricular tachycardia; Category 1: presence of 15 to 200 isolated events or pairs, or presence of supraventricular tachycardia or unsustained ventricular tachycardia; Category 2: presence of more than 200 events and presence of supraventricular tachycardia or sustained/unsustained ventricular tachycardia.

Supraventricular arrhythmias occurred in the form of isolated ectopic events and in pairs, not exceeding 20 events in nine of the 20 animals (45%). Periods of supraventricular tachycardia occurred in three cases (15%). Two of them were merely for a short duration, but the third presented twelve instances of moderate duration, approximately five seconds each. One dog that died during the 28-day follow up presented several escapes originated in the atrium during the 24-hour recording.

Ventricular arrhythmias occurred as isolated ectopic events and pairs, not exceeding 25 events in seven of the 20 animals (35%). Two (10%) presented a moderate number of occurrences, under 200 isolated events. Another two dogs (10%) presented a high number of occurrences, one with 7,578 ectopic events and the other with 6,418 isolated events in addition to 150 periods of unsustained ventricular tachycardia and two periods of sustained ventricular tachycardia, each lasting for approximately 30 minutes. No sinus pauses exceeding two seconds were recorded. The determination of the levels of cardiac troponin I was conducted for Category 2 dogs, revealing values of 2.14 ng/mL for the animal that presented large periods of sustained ventricular tachycardia.

**Fig 10:**
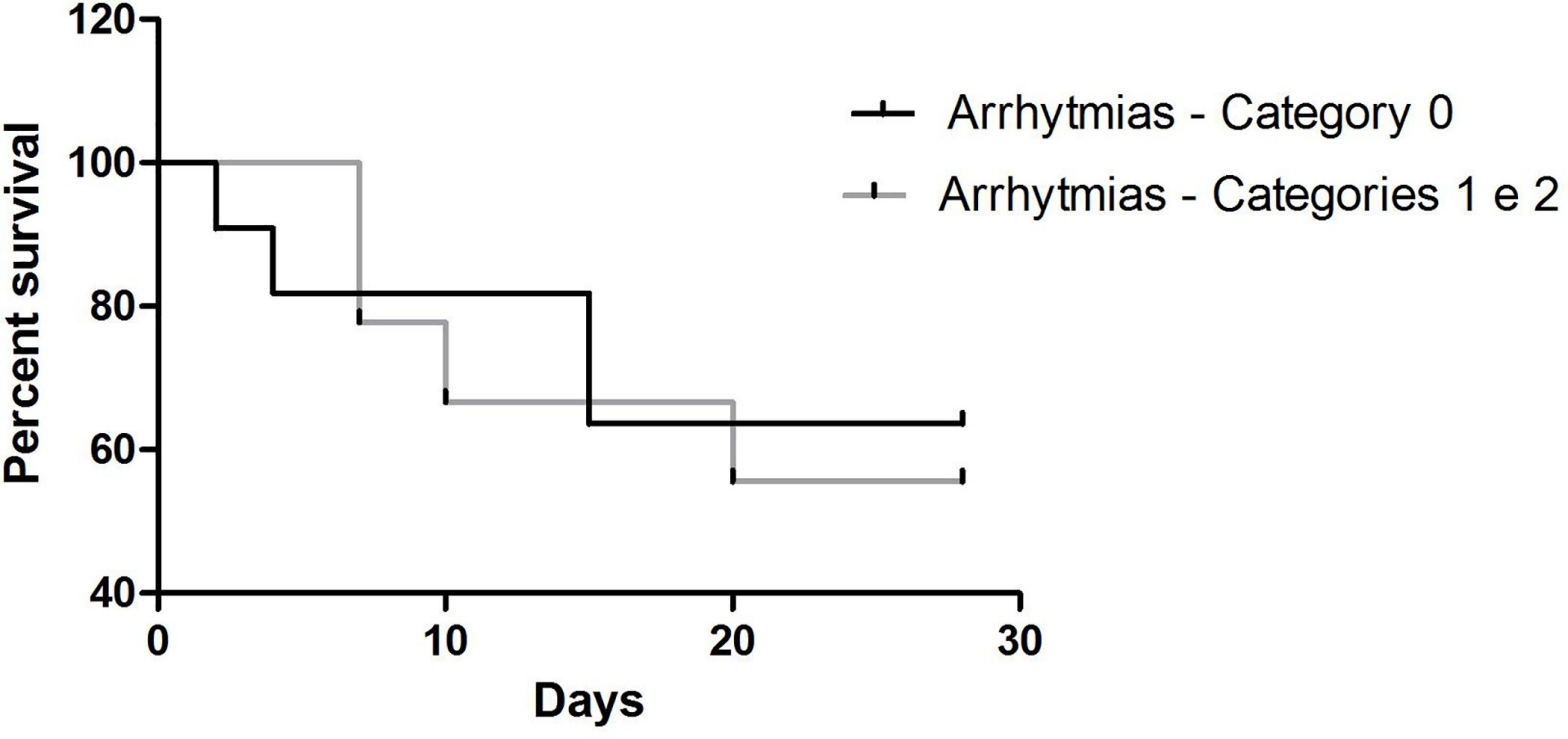
Survival analysis chart according to the presence of arrhythmias on the 24-hour Holter recording. The log-ranked test revealed a p-value of 0.45.

Of the 20 diseased animals, 8 died after the start of treatment and 12 survived. We did not perform an electrocardiogram after treatment, but mortality analysis (Fig 10) revealed that the presence of arrhythmias was not associated with increased mortality. However, subjectively, fewer dogs with arrhythmias at the beginning of the study died after the first seven days of treatment.

The time-domain indexes of heart rate variability were calculated, and the data is presented in Table 5. The mean values for SDNN, SDANN, SDNNi, RMSSD, and pnn50 for the sick group were much lower than the reference limits for the species, except for the number of NN intervals, which exceeded the normality thresholds. Statistically significant differences were observed for all parameters when comparing these results with the control group.

**Table 5:** Mean and standard deviation of time-domain heart rate variability (HRV) parameters in the sick and control groups. P values represent the results of the statistical tests.

HRV Parameters	Sick Group	Control Group	P value	Reference^a^
**Mean HR**	139.05 ± 28,58	86 ± 13,3	< 0,0001*^b^	81–105
**Minimal HR**	74,2 ± 18,53	38,5 ± 6,9	< 0,0001*^c^	30–58
**Maximal HR**	225,4 ± 25,3	249,1 ± 1,9	0,0111*^c^	235–250
**Mean NN**	467,15 ± 104,48	747 ± 117,6	< 0,0001*^b^	482–845
**SDNN**	83 ± 65	268 ± 74,6	< 0,0001*^b^	322,3 ± 137,4
**SDANN**	56,05 ± 37,3	168,3 ± 39,14	< 0,0001*^b^	148–202
**SDNN INDX**	57,55 ± 55,9	216,9 ± 74,28	< 0,0001*^b^	161–359
**NNs (x 10**^**3**^**)**	165,81 ± 36,73	101,47 ± 28,60	< 0,0001*^b^	79–130
**RMSSD**	55,2 ± 50	146,9 ± 45,5	0,0002*^c^	90–155
**TINN**	267,37 ± 113,38	648,88 ± 238,97	< 0,0001*^c^	-
**pNN50**	14,56 ± 20,1	55,87 ± 12,8	< 0,0001*^c^	48–69

^a^ Veterinary Cardiology Service—FMVZ UNESP Botucatu

^b^ t-test for independent samples

^c^ Mann-Whitney test.

*Statistical significance: p<0.05

## Discussion

The dogs included in this study presented results for the clinical, hematological and physical examinations similar to those reported in previous studies with dogs afflicted by cytopenic CME [6,24].

Considering that low hematocrit count and hypoproteinemia are conditions that not only decrease blood viscosity but also create a hyperdynamic state in the heart and that echocardiography exam performed on G1 dogs excluded possible heart diseases, not observing any valvar changes that could explain the murmurs, their occurrence may be caused this hyperdynamic state [25–28].

Increased capillary refill time (CRT) and systolic murmurs were observed in eight and 13 animals, respectively. The possible conditions associated with them are diverse, ranging from anemia to heart diseases [29,30].

The systolic blood pressure was significantly lower in the G1, but no hypotension (< 70 mmHg) or hypertension (> 150 mmHg) were observed in those animals despite the low values. A previous study observed hypertension in 12% of the dogs afflicted by CME, which was later found not to be a possible cause of the condition [31,32]. We believe that the lower blood pressure values in G1 than in G2, even though still within the reference values, are caused by the factors such as anemia, hypoalbuminemia, dehydration, possible cardiac hypocontractility and the release of inflammatory factors in the blood flow [33].

Few changes were observed in the analysis of the electrocardiographic parameters, mostly in the amplitude reduction of the P, R, and T waves. A hypothesis is that these changes may be caused by the reduction of intravascular volume secondary to dehydration, which may compromise electrical conduction due to the hydroelectrolytic changes that occur in this process[34].

Regarding ventricular repolarization, 50% of the dogs afflicted by cytopenic CME presented higher values for the T-wave, exceeding the rates described for animals afflicted by non-cytopenic CME [9]. This may be explained by the profile of anemia and heart disease. Studies with humans observed a T-wave inversion in 23% of patients suffering from chronic anemia [35]. There is evidence pointing towards a correlation between this inversion and areas of enhanced contrast in MRIs of the heart, one of the parameters used by cardiologists to identify myocarditis [36]. Therefore, this increase could be explained by the combination of a hematological condition and a heart disease.

Electrocardiographic alterations were observed in 45% of the cytopenic dogs, which is higher than the 35% observed in non-cytopenic dogs [9]. The types of arrhythmias themselves were similar.

Even though ventricular tachycardia is often associated with a poor prognosis in human cases of myocarditis caused by Chagas disease, and with increased risk of sudden death [37], the presence of major arrhythmias was not considered a risk factor for the animals who died in this study since the animal that presented severe arrhythmias survived for the entire 28-day follow up period.

The animals were evaluated only at the time of hospital admission and after confirmation of chronic Ehrlichiosis by the tests. One of the objectives of the study was to evaluate the arrhythmic potential of the disease, and since they were already in the chronic phase, we expected that there would already be changes in the electric conduction system and did not conduct any tests during the 28 days of treatment. However, clinical follow-up care was given in the hospital by the veterinarian responsible for the initial care, which allowed us to plot a survival curve for the animals.

However, we observed that the animals affected by chronic Ehrlichiosis presented arrhythmic events, but these arrhythmias were not associated with a worsened prognosis. It is important to note that, according to our results, chronic Ehrlichiosis predisposes the animal to arrhythmic events and patients afflicted by the condition should undergo an electrocardiogram.

The increased heart rate is a consequence of an exacerbated physiological stimulation of the autonomic nervous system and may occur due to cardiac or extracardiac changes, such as excitement, fever, hypovolemia, pain or as an early sign of heart failure [38,39].

The time-domain HRV indexes, even those that deal with long-term sympathetic stimulation, have shown differences during the circadian rhythm, including mean NN and SDNN. Short-term stimulation parameters have shown slight differences during respiratory movements, represented by the reduced SDANN and SDNNi indexes. The relationship with the parasympathetic indexes sensitive to variations between NN intervals, such as the pNN50 and RMSSD indexes, was also reduced.

HRV studies regarding heart diseases in dogs have also shown that higher sympathetic participation results in higher compensation in the heart and higher risk of congestive heart failure [40]. Decreases in these values were related to decompensation of heart disease in humans with idiopathic dilated cardiomyopathy [41]. Despite the lack of statistical significance, it was possible to observe a subjective relationship between low HRV indexes and the short-term mortality observed in this study, but a larger sample size is required to ascertain statistical significance.

The pnn50 index presented a significant difference between the groups, with G1 animals presenting lower values than G2. This fact may be due to the predominance of sympathetic ANS activity and, therefore, an imbalance of ANS activity during the chronic phase of the disease.

It has already been shown that histological alterations in the heart are common in cases of cytopenic CME. Mononuclear infiltrate, edema and necrosis are particularly common [12, 27, 28]. Histologic changes in the heart have been shown to be common in cases of cytopenic CME, with mononuclear infiltrates, edema and necrosis being particularly common. This study did not conduct histopathological analyzes in the animals that died, but we believe that myocarditis is a factor that may have contributed to the predominance of sympathetic activity observed in the holter examination, leading to an imbalance of the ANS activity. However, future studies are required analyzing the possible histopathological changes that may occur during the chronic phase of Ehrlichiosis, as well as correlating them with alterations in other complementary exams.

The occurrence of supraventricular tachycardia and the high number of ectopic events are not enough to cause increases in the concentrations of cardiac troponin I, and the dog with sustained ventricular tachycardia presented a significant increase. These findings suggest that severe tachyarrhythmias are harmful to the heart due to structural damage.

The rate of increased CKMB concentrations was higher than the one observed for cTnI. This can be attributed to the lower specificity of CKMB in relation to troponin I for myocardial detection since it can also be released as a result of muscle and renal injuries [45,46]. It should also be noted that the effect of minor increases in serum cTnI may be explained by the fact that transient or mild damage by cardiomyocytes may not generate a large and persistent release of cTnI. However, extensive damage may cause peak concentrations 2–4 hours after injury and cTnI concentrations may remain elevated within 24 hours of the event. Moreover, in mice, for example, a small compartment volume (due to its reduced size when compared to other mammals) contributes to a faster cTnI clearance in the serum, which indicates that sampling times to detect changes in Serum cTnI levels should be selected after considering species-specific variations in clearance rates [42–44].

According to an experimental study, the concentrations of these biomarkers increase when there is maximum myocardial damage, decreasing afterward. These increases show that there is, indeed, constant destruction, necrosis, edema and remodeling of myocardial fibers, probably secondary to progressive and intense infiltration of mononuclear cells, vasculitis, myocardial hemorrhage and immunoglobulin action [9]. However, the inconclusive survival analysis shows that, although the cardiac biomarkers were effective in detecting myocardial injury, they were not good prognostic indicators of survival.

Since NT-proBNP concentrations increases when there is a stress condition caused by volume/pressure overload on the cardiac chambers, we can assume that the dogs included in the study suffered from no such events. However, it is important to note that the concentration of NT-proBNP varies weekly between individual animals but may also vary considerably according to breed [46–48].

Biomarkers for myocardial injury, cTnI and CKMB were high in dogs with chronic CME in contrast with healthy dogs. No increases were observed in the concentrations of NT-proBNP. The concentrations of biomarkers cTnI and CKMB were high in the G1 animals, a fact that suggests the presence of myocardial injury during the chronic phase of the disease, but we did not carry out histopathological studies in the animals that died, which could contribute to the diagnosis of myocarditis. However, our results indicate that further studies are needed in animals with chronic Ehrlichiosis for histopathological analysis, since, according to the high concentrations of biomarkers observed in this study, there is a strong indication of myocardial injury. In addition, the histopathological analysis would exclude increases in concentrations caused by extracardiac factors.

Authors of several studies have established correlations between increases in the concentration of NT-proBNP with cardiomyopathies in dogs. However, the cardiac diseases analyzed in those studies were at stages involving cardiac remodeling with atrial volume overload, ventricular volume overload or, in some cases, both. This study did not observe evidence of remodeling since no animal presented valvar abnormalities in the echocardiogram performed to include animals in the study, which explains the results observed for the NT-proBNP marker [49–53].

## Conclusions

In the light of these results, chronic Ehrlichiosis predisposes the animals to arrhythmic events, but the presence their presence does not necessarily result in a worsened prognosis, varying according to each patient from mild to severe. The condition leads to autonomic imbalance with predominance of sympathetic activity, but factors such as anemia and hypoalbuminemia may also contribute to a greater imbalance in the activity of both branches of the ANS.

The concentrations cardiac biomarkers cTnI and CKMB were elevated in animals that could potentially be afflicted by myocarditis, a fact that must be confirmed through an histopathological examination and could be the focus of further research.

## References

[1] Dumler JS, Barbet AF, Bekker CP, Dasch GA, Palmer GH, Ray SC, et al Reorganization of genera in the families Rickettsiaceae and Anaplasmataceae in the order Rickettsiales: unification of some species of Ehrlichia with Anaplasma, Cowdria with Ehrlichia and Ehrlichia with Neorickettsia, descriptions of six new species combinations and designation of Ehrlichia equi and ‘HGE agent’ as subjective synonyms of Ehrlichia phagocytophila. Int J Syst Evol Microbiol. 2001; 1:2145–65. 10.1099/00207713-51-6-2145 11760958

[2] Everman JF, Sellon RK, Sykes JE. Viral, Rickettsial, and Chlamydial Diseases In: Greene C. Infectious Diseases of the dog and cat. Elsevier Saunders, Athens, GA, USA 2011.

[3] Harrus S, Waner T. Diagnosis of monocytotropic ehrlichiosis (Ehrlichia canis): An overview. Vet J. 2011;187:292–6. 10.1016/j.tvjl.2010.02.001 20226700

[4] Harrus S. Perspectives on the pathogenesis and treatment of canine monocytic ehrlichiosis (Ehrlichia canis). Vet J.2015; 203:239–40. 10.1016/j.tvjl.2014.12.008 25957922

[5] Mylonakis ME, Koutinas AF, Breitschwerdt EB, Hegarty BC, Billinis CD, Leontides LS, et al Chronic Canine Ehrlichiosis (Ehrlichia canis): A Retrospective Study of 19 Natural Cases. J Am Anim Hosp Assoc. 2004;40:174–84. 10.5326/0400174 15131097

[6] Hayes MA, Russell RG, Babiuk LA. Sudden death in young dogs with myocarditis caused by parvovirus. J Am Vet Med Assoc. 1979;174:1197–203. 438048

[7] Lobetti RG. Cardiac involvement in canine babesiosis. J S Afr Vet Assoc. 2005;76:4–8. 15900893 10.4102/jsava.v76i1.386

[8] Diniz PPVP, De morais HSA, Breitschwerdt EB, et al Serum Cardiac Troponin I Concentration in Dogs with Ehrlichiosis. J Vet Intern Med. 2008;22:1136–43. 10.1111/j.1939-1676.2008.0145.x 18638021

[9] Costagliola A, Piegari G, Otrocka-domagala I, et al Immunopathological Features of Canine Myocarditis Associated with Leishmania infantum Infection. Biomed Res Int. 2016 10.1155/2016/8016186 URL: www.ncbi.nlm.nih.gov/pmc/articles/PMC4930798/ 27413751 PMC4930798

[10] Vitt JP, Saunders AB, O'brien MT. Diagnostic Features of Acute Chagas Myocarditis with Sudden Death in a Family of Boxer Dogs. J Vet Intern Med. 2016;30:1210–5. 10.1111/jvim.13967 27163180 PMC5084738

[11] Elamm C, Fairweather D, Coope LT. Pathogenesis and diagnosis of myocarditis Heart 2012;98, 835–840. 10.1136/heartjnl-2012-301686 22442199 PMC12767484

[12] Krejci J, Mlejnek D, Sochorova D, Nemec P. Inflammatory Cardiomyopathy: A Current View on the Pathophysiology, Diagnosis, and Treatment. Biomed Research International. 2016;1–11. 10.1155/2016/4087632 PMC4921131 27382566

[13] Diniz PPVP De Morais HAS, Breitschwerdt EB Schwartz DS. Serum Cardiac Troponin I Concentration in Dogs with Ehrlichiosis. Journal of Veterinary Internal Medicine.2008; 22,1136–1143. 10.1111/j.1939-1676.2008.0145.x 18638021

[14] Haberman CE, Kang CW, Morgan JD, Brown SA. Evaluation of oscillometric and Doppler ultrasonic methods of indirect blood pressure estimation in conscious dogs. The Canadian Journal of Veterinary Research. 2006; 70, 211–217. 16850944 PMC1477936

[15] Brown S, Atkins CE, Bagley R, Carr A, Davidson M, Egner B, Elliot, Henik R. Guidelines for the Identification, Evaluation, and Management of Systemic Hypertension in Dogs and Cats. Journal of Veterinary Internal Medicine. 2007; 21, 542–558. 10.1892/0891-6640(2007)21[542:gftiea]2.0.co;2 17552466

[16] Garofalo NA, Neto FJT, Alvaides RK, Oliveira FA, Pignaton W, Pinheiro RT. Agreement between direct, oscillometric and Doppler ultrasound blood pressures using three different cuff positions in anesthetized dogs. Veterinary Anaesthesia and Analgesia. 2012;39, 324–334. 10.1111/j.1467-2995.2012.00711.x 22414262

[17] Wen B, Rikihisa Y, Mott JM, Greene R, Kim HY, Zhi N, Couto GC, Unver A, Bartsch R. Comparison of nested PCR with immunofluorescentantibody assay for detection of Ehrlichia canis infection in dogs treated with doxycycline, Journal of Clinical Microbiology. 1997;35, 1852–1855. 9196207 10.1128/jcm.35.7.1852-1855.1997 PMC229855

[18] Edwards NJ. Bolton’s Handbook of Canine and Feline Eletrocardiography. 2nd Edn W.B. Saunders Company, Toronto, ON, Canadá, 381 p.

[19] Tilley LP (Eds) Essentials of canine and feline electrocardiography. 3rd edn Lea and Febiger, Philadelphia, PA, USA:, 470 1992.

[20] Filippi LH (Ed) Eletrocardiograma na medicina veterinária, 1st Edn Roca, São Paulo, SP, Brazil, pp. 128–135.2001.

[21] Weiss DJ, Wardrop KJ, Schalm's Veterinary Hematology, 6th Ed, Wiley-Blackwell, 2010.

[22] Kaneko JJ, Harvey JW, Bruss ML, Clinical Biochemistry of Domestic Animals, 6th Ed, Academic Press 2008.

[23] Pino OV, Li EO, Alvarado AS, Fernández VP, Dávila RF, Gavidia CC. Determinación de los niveles séricos de enzimas cardíacas em perros adultos com enfermedad cardiovascular. Revista de Investigaciones Veterinarias del Peru. 2008;19:2,144–147.

[24] Sjöstrand K, Wess G, Ljungvall I, Häggström J, Merveille AC, Wiberg M, Gouni V, et al Breed differences in natriuretic peptides in healthy dogs. Journal of Veterinary Internal Medicine, 2014;28,2;451–457. 10.1111/jvim.12310 24495256 PMC4857989

[25] Oyama MA, Sisson DD, Cardiac troponin-I concentration in dogs with cardiac disease. Journal of Veterinary Internal Medicine. 2004;18:6,831–839. 10.1892/0891-6640(2004)18<831:ctcidw>2.0.co;2 15638266

[26] Frank JR, Breitschwerdt EB. A Retrospective Study of Ehrlichiosis in 62 Dogs from North Carolina and Virginia. J Vet Intern Med. 1999;13:194–201. 10.1892/0891-6640(1999)013<0194:arsoei>2.3.co;2 10357108

[27] Codner EC, Roberts RE, Ainsworth AG. Atypical findings in 16 cases of canine ehrlichiosis. J Am Vet Med Assoc. 1985;186:166–9. 3918976

[28] Sanches CDC. Estudo histopatológico das lesões viscerais da Erliquiose Monocítica Canina na fase crônica. São Paulo State University, Botucatu, SP, Brazil 2015 URL:https://alsafi.ead.unesp.br/handle/11449/144056. Last access: 01/11/2017.

[29] Ware WA. Diagnostic tests for the Cardiovascular System In: Nelson RW, Couto CG. Small Animal Internal Medicine. Elsevier Saunders, Davis, CA, USA p.13–53.

[30] Fowler NO, Holmes JC. Blood viscosity and cardiac output in acute experimental anemia. J Appl Physiol. 1975;39:453–6. 10.1152/jappl.1975.39.3.453 1176411

[31] Spotswood TC, Kirberger RM, Koma LMPK et al Changes in echocardiographic variables of left ventricular size 119 and function in a model of canine normovolemic anemia. Vet Radiol Ultrassound. 2006;47:358–65. 10.1111/j.1740-8261.2006.00154.x 16863054

[32] Bavegems VC, Duchateau L, Polis IE, et al Detection of innocent systolic murmurs by auscultation and their relation to hematologic and echocardiographic findings in clinically normal Whippets. J Am Vet Med Assoc. 2001;238:468–71. 10.2460/javma.238.4.468 21320016

[33] Szatmári V, Van leeuwen MW, Teske E. Innocent Cardiac Murmur in Puppies: Prevalence, Correlation with Hematocrit, and Auscultation Characteristics. J Vet Intern Med. 2015;29:1524–8. 10.1111/jvim.13632 26415555 PMC4895663

[34] Diniz, PPVP. Miocardite em cães com Ehrlichiose Monocítica Canina. Thesis, PhD of Veterinary Medicine—São Paulo State University, Brazil,2006.

[35] Saltykova MM, At'kov O, Karlin EK, Zaruba A, Dmitriev AA, Kukharchuk VV. Increased QRS voltage during dehydrating. Terapevticheskii arkhiv. 2007;79(4), 18–23. 17564013

[36] Stanojević M, Stankov S. Electrocardiographic changes in patients with chronic anemia. Srpski Arhiv Za Celokupno Lekarstvo. 1997;126, 461–466. 9921020

[37] Nucifora G, Miani D, Di chiara A, Piccoli G, Artico J, Puppato M, Slavich G, De biasio M, Gasparini D, Proclemer A. Infarct like Acute Myocarditis: Relation Between Electrocardiographic Findings and Myocardial Damage as Assessed by Cardiac Magnetic Resonance Imaging. Clinical cardiology. 2013;36, 146–152. 10.1002/clc.22088 23280562 PMC6649633

[38] Távora MZP, Mehta N, Silva RMF, Gondim FAA, Hara VM, De Paola AAV. Characteristics and Identification of Sites of Chagasic Ventricular Tachycardia by Endocardial Mapping. Arquivo Brasileiro de Cardiologia. 1999;72:4,463–474. 10.1590/s0066-782x1999000400006 10531690

[39] Hadlock DJ. Sinus tachycardia In: Tilley LP, Smith JR, (Eds). The five minute veterinary consult: canine and feline, 2nd edn Willians & Wilkins, Philadelphia, PA, USA, pp. 380.

[40] Taralov ZZ, Terziyski KV, Kostianev SS. Heart rate variability as a method for assessment of the autonomic nervous system and the adaptations to different physiological and pathological conditions. Folia medica.2015;57,3–4:173–180. 10.1515/folmed-2015-0036 27180343

[41] Pereira YM, Wooley R, Culshaw G, French A, Martin M. The vasovagal tonus index as a prognostic indicator in dogs with dilated cardiomyopathy. Journal of Small Animal Pratice. 2008;49, 587–592. 10.1111/j.1748-5827.2008.00654.x 19006490

[42] Goldman LE, Sahlas DJ, Sami M. A case of thyrotoxicosis and reversible systolic cardiac dysfunction. The Canadian Journal of Cardiology. 1999;15, 811–814. 10411619

[43] Lang H, Wurzburg U. Creatine kinase, an enzyme of many forms. Clin Chem. 1982;28:1439–47. 7044614

[44] Tharwata M, Al-Sobayila F. The cardiac biomarkers troponin I and CK-MB in nonpregnant and pregnant goats, goats with normal birth, goats with prolonged birth, and goats with pregnancy toxemia. Theriogenology. 2012; 78: 1500–1507. 10.1016/j.theriogenology.2012.06.013 22980083

[45] Dunn ME, Colucio D, Hirkaler G, Mikaelian I, Mikaelian R, Lipshultz SE, et al The Complete Pharmacokinetic Profile of Serum Cardiac Troponin I in the Rat and the Dog. Toxicol Sci. 2011;123:368–73. 10.1093/toxsci/kfr190 21775726

[46] Sjöstrand K, Wess G, Ljungvall I, Häggström J, Merveille A-C, Wiberg M, et al Breed differences in natriuretic peptides in healthy dogs. J Vet Intern Med. 014;28:451–7. 10.1111/jvim.12310 24495256 PMC4857989

[47] Kellihan HB, Oyama MA, Reynolds CA, et al Weekly Variability of plasma and serum NT-proBNP measurements in normal dogs. J Vet Cardiol. 2009;11:593–7. 10.1016/j.jvc.2009.03.003 19395335

[48] Omland T, Lemons JA, Sabatine MS, Stepien RL. Sensitive Cardiac Troponin T Assay in Stable Coronary Artery Disease. N Engl J Med. 2009;361:2538–47. 10.1056/NEJMoa0805299 19940289 PMC2997684

[49] Chetboul V, Serres F, Tissier R, Lefebvre HP, Carlos Sampedrano C, Gouni V, et al Association of plasma N-terminal pro-B-type natriuretic peptide concentration with mitral regurgitation severity and outcome in dogs with asymptomatic degenerative mitral valve disease. J Vet Intern Med. 2009;23:984–94. 10.1111/j.1939-1676.2009.0347.x 19572913

[50] Serres F, Pouchelon JL, Poujol L, et al Plasma N-terminal pro-B-type natriuretic peptide concentration helps to predict survival in dogs with symptomatic degenerative mitral valve disease regardless of and in combination with the initial clinical status at admission. J Vet Cardiol. 2009;11:103–21. 10.1016/j.jvc.2009.07.001 19850546

[51] Noszczyk-nowak A. NT-pro-BNP and troponin I as predictors of mortality in dogs with heart failure. Pol J Vet Sci. 2011;14:551–6. 22439324 10.2478/v10181-011-0082-z

[52] Singletary GE, Morris NA, Lynne o’sullivan M, Gordon SG, Oyama MA. Prospective evaluation of NT-proBNP assay to detect occult dilated cardiomyopathy and predict survival in Doberman Pinschers. J Vet Intern Med. 2012;26:1330–6. 10.1111/j.1939-1676.2012.1000.x 22998090

[53] Kobayashi K, Hori Y, Chimura S. Plasma N-Terminal Pro B-Type Natriuretic Peptide Concentrations in Dogs with Pulmonic Stenosis. J Vet Med Sci. 2014;76:827–31. 10.1292/jvms.13-0554 24561377 PMC4108765

